# Ghost hunting in the nonlinear dynamic machine

**DOI:** 10.1371/journal.pone.0226572

**Published:** 2019-12-19

**Authors:** Jonathan E. Butner, Ascher K. Munion, Brian R. W. Baucom, Alexander Wong

**Affiliations:** 1 Department of Psychology, University of Utah, Salt Lake City, Utah, United States of America; 2 Department of Psychology, California State University at Chico, Chico, California, United States of America; Beijing University of Posts and Telecommunications, CHINA

## Abstract

Integrating dynamic systems modeling and machine learning generates an exploratory nonlinear solution for analyzing dynamical systems-based data. Applying dynamical systems theory to the machine learning solution further provides a pathway to interpret the results. Using random forest models as an illustrative example, these models were able to recover the temporal dynamics of time series data simulated using a modified Cusp Catastrophe Monte Carlo. By extracting the points of no change (set points) and the predicted changes surrounding the set points, it is possible to characterize the topology of the system, both for systems governed by global equation forms and complex adaptive systems. RESULTS: The model for the simulation was able to recover the cusp catastrophe (i.e. the qualitative changes in the dynamics of the system) even when applied to data that have a significant amount of error variance. To further illustrate the approach, a real-world accelerometer example was examined, where the model differentiated between movement dynamics patterns by identifying set points related to cyclic motion during walking and attraction during stair climbing. These example findings suggest that integrating machine learning with dynamical systems modeling provides a viable means for classifying distinct temporal patterns, even when there is no governing equation for the nonlinear dynamics. Results of these integrated models yield solutions with both a prediction of where the system is going next and a decomposition of the topological features implied by the temporal dynamics.

## Introduction

Advancements in machine learning are frequently utilized for an extensive array of applications, from pattern identification and robotics to classification [1, 2] and are even harnessed for traditional statistical analyses [3]. Statistical learning generally serves two purposes: 1) prediction of a response variable (i.e., classification), and 2) to address inferential questions–identifying the relationships between sets of variables [4]. Machines generate a prediction algorithm but do not generally provide the decomposition of the linear, or nonlinear, relationship between various predictors within the analysis–even those that focus on inference. When the goal is categorization or outcome prediction, the decomposition of relationships building the prediction is of minimal importance.

When machine learning approaches are applied within the context of inferential dynamical systems analyses, the results provide a rich set of information about both prediction and decomposition of the different temporal patterns reflected in the data set. Dynamical systems methods are a collection of approaches for characterizing complex linear and nonlinear patterns of change over time and cover many concepts from complexity, dimensionality and chaos, to the identification of set points, bifurcation, and phase transitions [5]. Machine learning and dynamical systems integrations include applications in robotics [6, 7] and online processing of one or multiple simultaneous channels, such as with EEG data [8]. These integrations of machine learning and dynamical systems theory have allowed both real-world performance improvements like replication of humanoid kinematics [9] and for statistical applications such as identification of non-stationarity in time series [10].

Many systems-based machine learning approaches allow for prediction while also quantifying the global pattern of temporal change [11]. However, not all systems conform to a single equation (i.e., a global description); in these instances, researchers are seemingly limited to merely a prediction model. This paper explores the use of machine learning approaches for detecting qualitatively distinct patterns of change over time (i.e., localized descriptions) for understanding complex nonlinear change processes. We do so by extending the traditional prediction applications of machine learning algorithms to characterize the topology underlying a multivariate time series, predicting qualitatively different dynamics of change over time, while also predicting the next value of the time series. Since dynamic systems models generate parameter estimates that can be used to decompose the temporal qualities of a system (e.g., stability, set points, etc.), it is possible to extract these qualities from the different temporal patterns in a generated machine. This approach has the advantage of not requiring a specific global description of the system while simultaneously providing local and global interpretation. This paper explores how seeking set points and the stability around those set points can decompose complex dynamical systems-based machine learning solutions. To exemplify this approach, we utilize random forest regression [12] on results from data generated by the cusp catastrophe–an equation that can generate complex nonlinear behavior over time. Further, we then exemplify the technique on accelerometer data from a mHealth data set. It is important to note that while we utilize random forest regression, the proposed technique exemplifies the extraction of system dynamics and can be integrated with many algorithmic approaches.

### Systems-based machine learning

Complex physical systems follow canonical dynamic topologies, [5] which conforms to differential and difference equation descriptions of data over time. Many systems models, including some machine learning integrations, capitalize on these well-known equation forms to characterize systems topology in many areas of research, including financial, bio-medical, and ecological (see [13–15] for examples). Bongard and Lipson [16] and Schmidt and Lipson [17], produced a method to identify a set of equations that best characterize data from a set of possible equation forms. Built from genetic programming, the process identifies nonlinear differential equations, seeking parsimony in the complexity of the model, number of terms, and model accuracy. Alternatively, Brunton et. al. [18] suggested a procedure for finding governing equations using sparse identification. One of the primary advantages of both of these systems-based machine learning integrations is that they can identify linear and nonlinear dynamic equation forms. They seek a differential equation form, yielding a global description of the change processes for systems that conform to a topology described by a single equation. While these solutions can describe complex topology, they are not inherently flexible enough to deal with many qualitative changes–either smooth or catastrophic–in the dynamic if not directly articulated in the tested equation. For instance, many global governing equations, such as those for a damped oscillator, do not account for bifurcations, or multi-stability [19].

Some systems are instead described as complex adaptive systems [20]. Such systems are thought to locally conform to the properties of differential and difference equations, but globally generate patterns that are more difficult to describe with a finite set of equations due to autocatalytic and adaptive effects [21]. The techniques described above are well suited to seeking equations that characterize the temporal dynamics of complex physical systems, but are less useful for complex adaptive systems because their global patterns are not well-described by one governing equation [22]. Developing techniques that are flexible enough to predict both where the adaptive system is going and also what the topology of the system is will help those who are interested in both predictive (i.e., identification of different temporal dynamics) and inferential questions (i.e., characterization of the temporal dynamics). For instance, research questions for those who utilize machine learning for biomedical data, especially those interested in interventions, are likely to involve both temporal dynamics (e.g., are symptoms improving/deteriorating?) and qualitative changes in the dynamics (e.g., did the dynamics change when the intervention was given?).

It is possible to generate equations that describe the topological features of the dynamics of complex adaptive systems [23]. Systems share local dynamic qualities that can be characterized using differential and difference equations. Adaptive systems do not conform to a single governing equation but instead have the capacity to evolve through multiple dynamic patterns, each with its unique array of topological features. By focusing on identifying the type, location, and strength of different topological features of the system, it is possible to generate both a local and global description without having to specify global equation form. In some cases, identifying the global governing equation is the primary interest; however, in many cases, extracting the topological features implied by the local governing equations adds additional insight, and such extractions are invaluable in cases where there is no governing equation.

### Extracting dynamical systems properties using machine learning

To illustrate the principles of integrating dynamic systems modeling and machine learning techniques, we use a difference equation to quantify the topological features of dynamic systems. We elected to use a difference equation representation for ease of presentation and to minimize the complexity of the dynamic systems model itself. It is important to note that there are other mathematical representations of topological features of dynamic systems, such as differential equations, that can be integrated with machine learning techniques. In a difference score representation of the topology of a dynamic system, the discrete difference in some variable is predicted by the current value of the same variable and any other variables that might predict differences in the underlying dynamic in some unknown or known function *F*.

$$Y_{t+1}-Y_{t}=F(Y_{t},C_{t})$$ (1)

In Eq 1, Y_t_ is the current value of the variable of interest Y_t+1_ is the next immediate value in Y. C is the set of variables that quantify the likelihood of the system being in one or more stable relationships between the difference in Y from time = t to time = t+1 and current Y, sometimes called control parameters [24]. Under complex physical systems, the function *F* has an identifiable equation form. Under adaptive systems, *F* may have multiple solutions and would not necessarily conform to a single equation. It is also possible to expand the model beyond a single outcome (i.e., Y) by expressing the equation as matrices instead of as scalars. In this case, a set of discrete differences in each variable at each point in time is predicted by the set of current values of those same variables and a set of potential control parameters.

Eq 1 can be implemented in a machine learning framework when *F* is unknown and may have multiple solutions. We utilize machine learning tools that capture nonlinear forms of the system to allow for a flexible form of *F*. To illustrate this approach, we demonstrate how a Random Forest Regression [12, 25] simultaneously tests many different models where the relationship between the outcome with Y and C are different above and below cutoffs in Y and C. Random forest regression is an extension of random forest classifiers, where each tree in the random forest depends on the values of a random independently sampled vector and trees in the forest have the same distribution [12]. Random Forests are used for prediction in many areas of science and are popular because there are minimal parameters to tune, and they function well with small sample sizes [4, 26]. Alternatively, Support Vector Machines (SVM’s) can be used for this purpose. SVMs follow the classic, rather than extreme machine learning features, where training data are mapped to global features through nonlinear functions [27], then standard optimization methods are applied to minimize training error and maximize the separation between feature spaces for the classes [28, 29]. Support Vector Regression is an adaption of the general SVM framework for use with regression models with continuous outcomes [30]. SVM regressions identify vectors in Y and C whereby the relationships between Y and C on the outcome are different on opposing sides of vectors. Both random forest and SVM regression (and others) can approximate nonlinear relationships, including interactions between Y and C and complex nonlinear forms of Y and C with the outcome. Standard applications of Random Forest and SVM regressions, while excellent at prediction, are difficult to interpret with respect to underlying relationships between variables and outcomes. However, when Random Forest Regression or SVM Regression are combined with difference equation representations of system’s dynamics, the underlying topological properties are recaptured by the solution. This approach is able to emulate systems governed by global equations and complex adaptive ones. Harnessing difference score models allows for management of both sparse and less-sparse nonlinear dynamic systems, and extension involving other machines, including extreme learning machines.

The goal of the proposed technique is to identify the set points of the system, and the behavior around those set points in order to characterize the topological features of the system. Complex (adaptive and physical) systems can be broken down into basins, where the expected behavior within a basin conforms to a similar change pattern [22]. Canonical examples include attractors, repellers, saddles, cycles, and strange attractors where values move towards, away, and around a relative point in the system (see [5] for a review of topological systems). Set points and the behavior around the set points correspond to the different basins of the system and describe change processes within the basin. The distinction between physical and adaptive systems can be characterized by the relationship amongst the various basins. Systems governed by global equations have behaviors both within and across basins that are characterized by a set of differential or difference equations. Adaptive systems, on the other hand, can have changing forms for the basins and between basins. However, the basins themselves still have a difference equation representation.

When there is a difference equation description as depicted in Eq 1, the equation implies the existence of set points. A set point is a set of values on the predictor side of the equation where no change occurs. All of the canonical systems behaviors have at least one set point. What distinguishes attractors from repellers, for example, is what the behavior of the system does relative to the set point. In the context of Eq 1, set points occur where the left side of the equation is zero and can be generalized to machine learning approaches as places the machine would predict zero change. To determine where a machine learning model predicts set points, a grid of data is built as a function of possible values of Y and C, and the grid is used to generate predicted values. Current applications of machine learning that utilize continuous outcomes (e.g., SVM Regression and Random Forest Regression) can be used to identify set points with no modification as these machines can all generate predicted values.

However, it is possible for predicted values to diverge from zero and still not indicate a meaningful difference from 0. To determine the true difference from 0, some criteria of meaningful versus negligible variability must be utilized, criteria which machine learning does not currently assess. To assess these criteria, we utilize quantile regression because a) it integrates with some support vector machines and random forest models [31, 32], and b) it allows for extracting measures of variability. More specifically, we utilize Random Forest Regression to predict where the set points are via identifying where predicted change is 0. Quantile regression additionally allows for establishing benchmarks for meaningful differences from 0 by creating pseudo-confidence intervals that we use to determine which predicted values are not different from 0 (i.e., which confidence intervals do not include 0). These measures of variability can be used to identify bounds for set points, operationalized as when zero falls between two predicted quantiles, one above and one below the 50^th^ quantile.

Once the set points are extracted, the dynamic behavior around each set point is identified by generating predicted values using values higher and lower on Y only (C values are held constant) and determining the direction of change over time in the system relative to the set point. If the predicted changes imply that the system is approaching the set point, it is attractive. If the predicted changes imply that the system is diverging from the set point, it is repulsive. It is also possible to identify transient points that approach in one direction and diverge in another [33]. Transient points represent places where the system slows down in the rate of change over time, but that are not topologically stable. Table 1 contains the steps of the procedure. Code for the integration of quantile regression with Random Forest Regression is provided in S2 File. This general approach of integrating equation-based descriptions of the topological features of dynamic systems and machine learning approaches can be extended to other machines and is intended to serve as a theoretical extension to machine learning, rather than as a single limited application. Through this approach, the prediction generated by Random Forests is preserved while also identifying the qualitative changes in the topology of the system–giving insight into the system for those who are simultaneously interested in the relationships underlying the topology.

**Table 1:** Proposed analytic steps.

Step 1.	*Generate the outcome(s)*. Taking each of the time series, calculate a lead of the variable(s) to be treated as the outcomes. In the provided code, this is done through the Hmisc package [34]. Differences are then calculated using lead minus current.
Step 2.	*Make cross-validation samples*. Split the data into cross-validation samples for training and testing. This was not done in the cusp example since comparisons were made to a known model.
Step 3.	*Generate the random forest models*. Conduct a Random Forest Regression (or SVM regression) model using the difference as the outcome on training data. The current value of the same variable as a predictor along with other variables. Other variables can be current values from other simultaneous variables such as was done in the real-world data example or variables that might function as control parameters for differentiating the qualitative dynamics. In the provided code this was done through the RandomForestSRC package [35].
Step 4.	*Establish the generalizability of the model*. Cross validate the model on testing data to determine the generalizability of the model. For the cusp model, this was replaced with examining Cobb’s R-square for the cusp catastrophe using the cusp package since the true model was known [36]. Note that the approach for the cusp model was applied to the set points which are generated in step 6.
Step 5.	*Generate quantile models*. Conduct Quantile Regression on the same data source extracting out quantiles above and below the 50^th^ percentile. For the cusp model, the 40^th^ and 60^th^ were extracted. For the real-world example, all even integer quantiles were extracted. This was done through the RandomForestSRC package [35].
Step 6.	*Identify set points*. Determine which combination of predictor values are likely set points. To do so, build a grid of different value combinations from the set of predictors. Using the Quantiles from the previous step, generate Quasi- Confidence Intervals predicted for each coordinate. The coordinate is a likely set point if the confidence interval includes a predicted change of zero. For more than one simultaneous equation, the coordinate must predict a confidence interval, including zero for all the equations. This can be done for a specific quantile range (i.e., the cusp example) or using many different quantile ranges (i.e., the real-world example).
Step 7.	*Identify the dynamic associated with each Set Point*. Using the random forest regression model(s), generate the predicted change for values in the predictor set that are slightly above and below each set point. Control parameters–the variables whose changes are not being treated as an outcome in any models–are held constant to the set point value. Attraction is identified when the predicted changes are towards the set point (positive change when below and negative change when above). Repulsion is identified when the predicted changes are away from the set point (negative change when below and positive change when above). Transient points are when the predicted changes on opposing sides of the set points are in the same direction. It is also possible to observe angular motion associated with cycles (neither towards nor away). The unit difference above and below should be relatively small to ensure the evaluation of the dynamic is within the basin associated with the set point. We utilized between half and a whole unit used to generate the grid in step 6.
Step 8.	*Visualize the Set Points and their related dynamics*. There is no fixed way to represent this step. For the cusp model, we utilized the rgl package [37] and the car package [38] to graph the set points and the R-square values, respectively. For the real-world example, we utilized the ggplot2 package [39] to generate a heatmap of the likely set points from step 6 and a predicted vector plot to represent step 7.

By identifying the set points and the local behavior around the set points we rebuild the dynamics that the machine learning model captures regardless of whether the model is depicting a system governed by global equations or an adaptive system.

### Exploring topology through the cusp catastrophe

To test the logic of extracting set points and the behavior from a systems-based machine integration, we utilize data from a complex physical system known as the cusp catastrophe model [40]. The cusp catastrophe model serves as an excellent example of systems, due to a complexity in topology, while maintaining a relatively simple equation form:

$$F(Y,A,B)=Y^{4}+BY^{2}+AY$$ (2)

**Fig 1:**
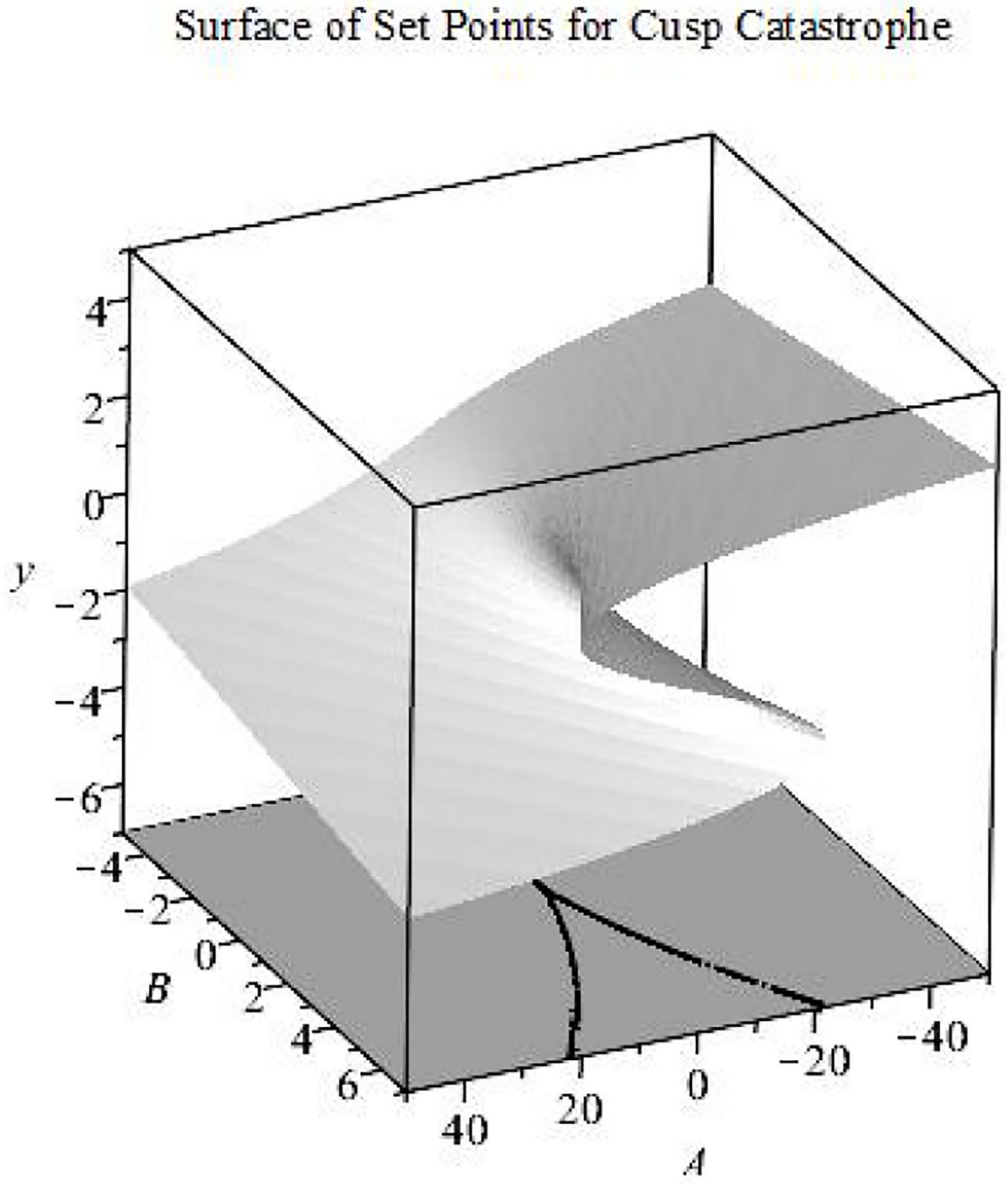
Set points implied by the cusp catastrophe surface. The cusp catastrophe has two control parameters that capture when data will illustrate bimodality and divergence. Catastrophe models have five flags in total, all of which can be found in the cusp catastrophe model [44].

The cusp catastrophe is an ideal system to explore because it contains both smooth (A–asymmetry control parameter) and abrupt changes (B- bifurcations control parameter) in the topology of the underlying phenomena [41] and can capture multimodality where more than one stable pattern through time is possible [42, 43]. The Cusp Catastrophe can be represented as a phase-space (Fig 1) of possible set points given values of parameters A, B, and Y from Eq 2. The equation (Eq 2) creates a twisted topological surface representing a system with many possible set points, which includes both attractors and repellers. Both A and B from Eq 2 function as control parameters (C in Eq 1) indexing the smooth or abrupt nonlinear change in the topology, respectively.

The A and B control parameters also dictate the likelihood of the set points and the stability of those set points. When B is negative, A dictates the location of the set point from low to high Y (as A increases). When B is positive, a fold appears in the surface such that, within a limited range of A, there are three set points instead of only one. The middle set point is repulsive, separating two points of attraction. Fig 1 maps how asymmetry and bifurcation interact to create folds, allowing for uni-stability and multi-stability within the same system.

Cusp catastrophes have been identified in health contests (e.g., grip strength during aging) with a gene acting as immune response regulator (interleukin-6) serving as an asymmetry factor and executive function serving as a bifurcation factor [45]. Current machine learning techniques can identify if this underlying governing equation (Eq 2) best describes the system–that the data is consistent with a cusp catastrophe. However, we are also interested in the case where the dynamic may not follow a specific form, such as would happen in complex adaptive systems. For this reason, we focus on characterizing the local behaviors of the system–the specific strength and location of topological features–by capturing set points, and the stability around those set points. For many applications, extracting what features exist, the stability and location of said features, and the ability to explore individual differences in the features are likely to become increasingly important. As machine learning is harnessed for wider statistical applications, due to its ease in handling sparse and large data sets, machine learning is ever-adapting, employed far beyond just categorization [46]. Thus, we seek an accessible analytic procedure that re-captures the nonlinear aspects of dynamic systems, while providing characterizations of the topological features of interest (such as set points).

### Simulating data from the cusp catastrophe

We simulated time series data from a modified form of the cusp catastrophe model (excluding some constants; [47]).

$$Y_{t+1}-Y_{t}=-Y^{3}+B_{t}Y_{t}+A_{t}+e_{t}$$ (3)

A and B are the control parameters described above (likelihood of set points) that dictate when the cusp shows uni-stability or multi-stability. The e_t_ term represents perturbations (or transient error) in the system. Perturbations are a fundamental part of dynamical systems, assumed to be present in all open systems, and essential to differentiating stable and unstable states [48]. Our code to simulate data from Eq 3 in R is provided in S1 File.

We ran a Monte Carlo simulation of time series of length 250, 500, and 1000 and varying amounts of perturbation error following a Gaussian distribution (the magnitude of the standard deviation in predicting change using half a standard deviation, a full standard deviation, and one and a half standard deviations) using 500 samples for each condition. The Gaussian distribution was chosen to be consistent with statistical modeling assumptions. We used a sample size doubling as standard practice in machine learning applications is to cross-validate with at least two samples, sometimes using half of the data to train the model and another half to test the model. Thus, the doubling matches these splits. We used the simulator defaults of sampling A and B from a uniform distribution between -.5 and .5. It is possible to instead fix A and B to a constant, allowing for exploration of a specific space in the surface rather than the entire range of the cusp model. This option is not used in our simulations and provided in the code for users who wish to further explore the Cusp Catastrophe model.

For the random forest model, we utilized the RandomForestSRC package in R that integrates quantile regression with random forest models applied to each time series separately [35]. Taking the data produced from the cusp catastrophe simulator we predicted the discrete difference of Y from the current value of Y, A, and B. Further, we extracted out the predicted values for the 40^th^ and 60^th^ quantiles demarking a set point when the quantiles included zero within the range (set point was identified when 40^th^ quantile < = 0 < = 60^th^ quantile).

All set points were then examined by generating predicted values of Y at the same value of A and B, but where Y was slightly above or below the set point (based on the metric chosen for Y, we used +/- .05). The set point was determined to be attractive if both predicted changes were towards the set point, repulsive if both were away from the set point and transitive if one was towards and one was away.

### Quantifying recovery and depiction of the cusp catastrophe

Determining how well the random forest model in conjunction with our focus on set points and stability recaptured the Cusp Catastrophe is difficult since the fold in the Cusp model makes characterizing model fit statistically ambiguous. We therefore relied on Cobb’s pseudo-R square [49] to represent the fit of the catastrophe model, calculated within the ‘cusp’ package in R. Cobb’s pseudo-R square is a modified form of R square that takes into account the cusp by integrating the calculation with a likelihood of being on each side of the cusp, combining results above and below the fold. Further, we provide a linear R-square multiple as a function of Y, A, and B, which accounts for the set points ignoring the fold. Both Cobb’s pseudo-R square and linear R square are zero when applied to the raw data generated by our cusp catastrophe simulator. Thus, applying the R square calculations to the set points identified through the proposed procedure becomes an indicator of the extent to which the dynamics of the cusp catastrophe are properly recovered by the machine and in our focus on set points.

## Results from cusp simulation

**Fig 2:**
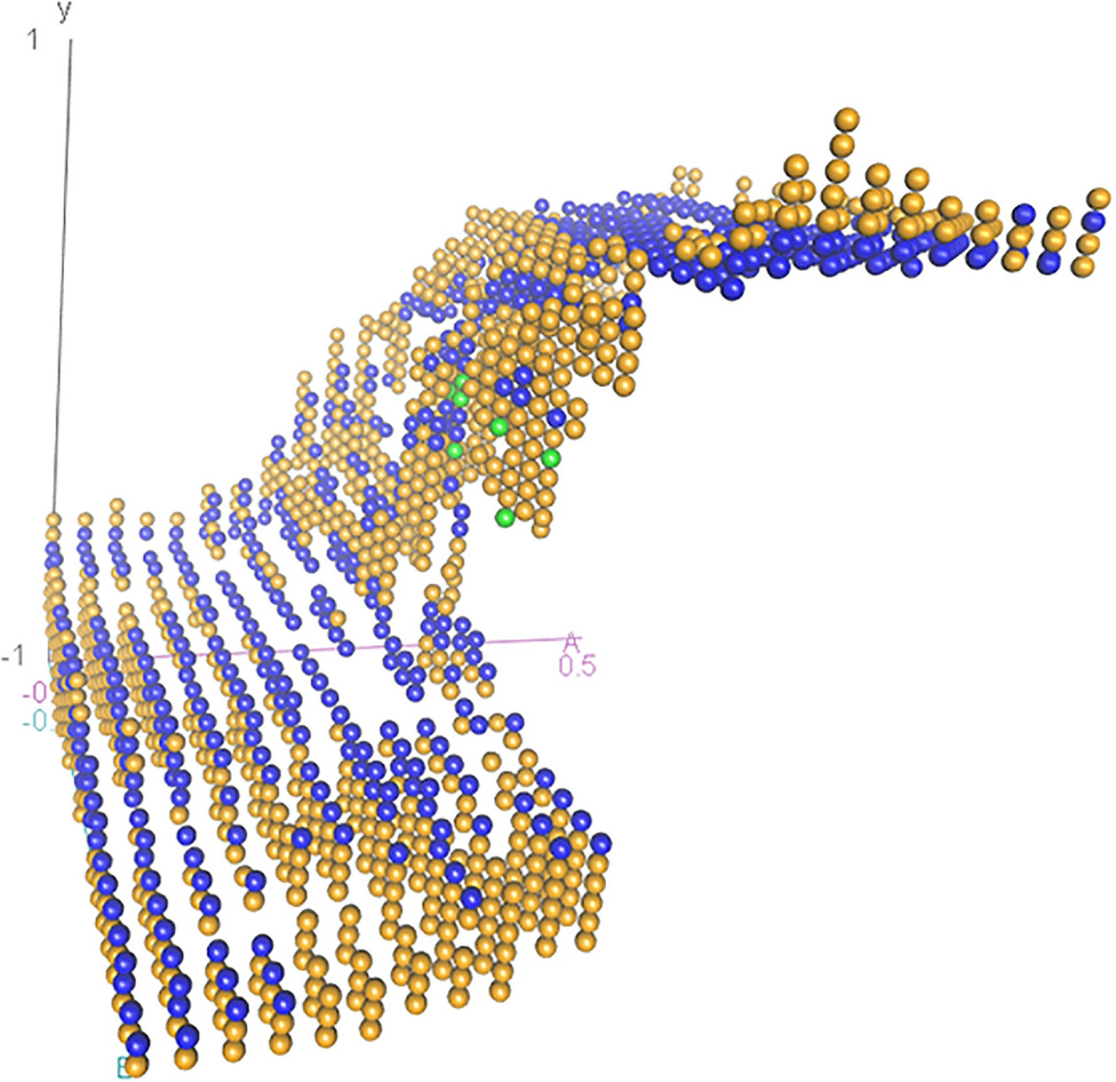
Set points from a random forest regression model on the same data set as Fig 3. Set points were chosen by seeing if predicted change of zero was between the 40^th^ and 60^th^ quantiles. The slope at each set point was determined by examining the predicted change in Y at values of Y 0.1 above and below the set points. Attractors (convergence toward the set point) are blue, repellers (divergence away from the set point) are green, transient points (attractive in one direction and repulsive in another) are tan.

**Fig 3:**
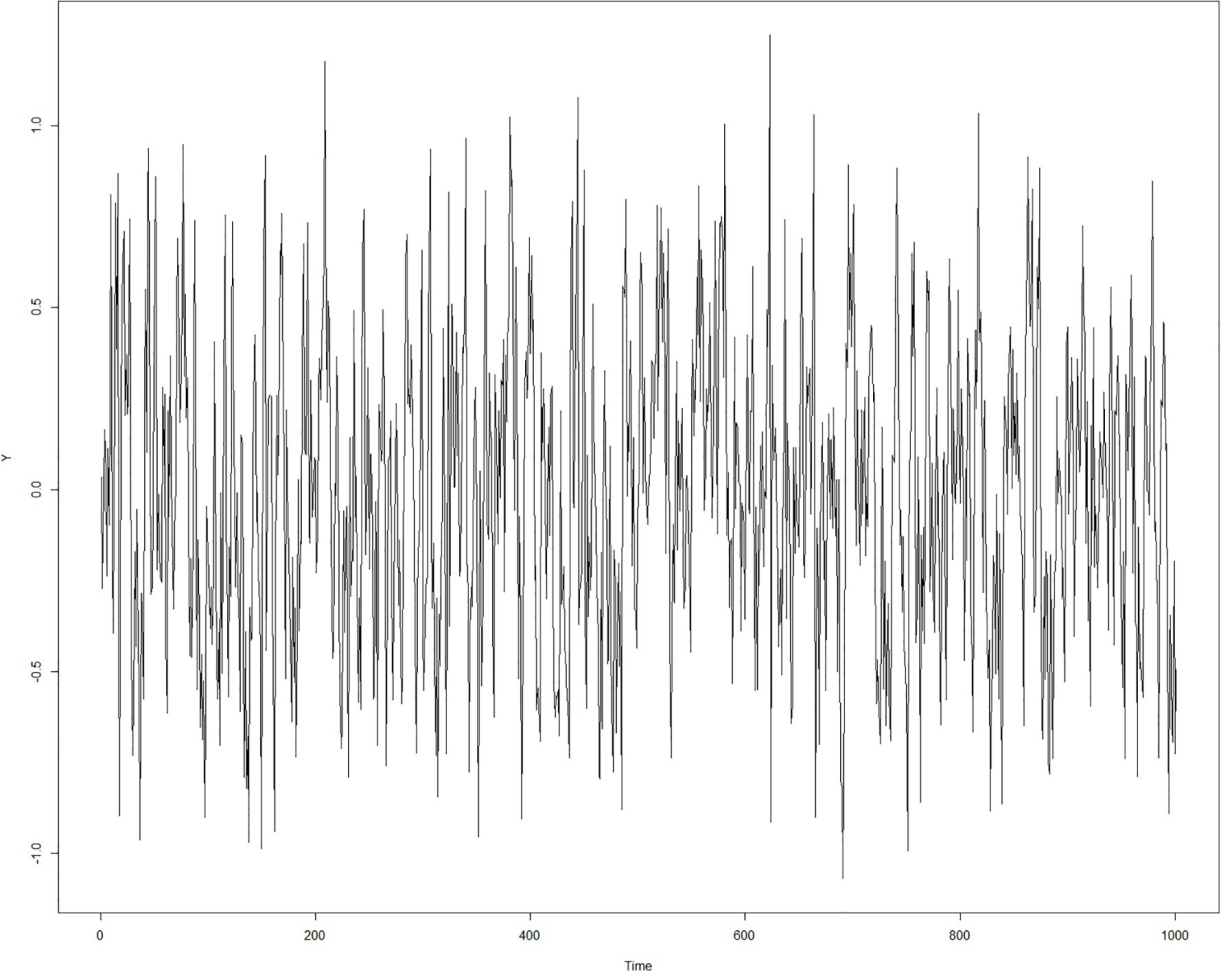
A time series of 1000 data points over time following Eq 2. A and B were drawn from a uniform distribution with a range of -0.5 to 0.5 at each point in time with perturbations represented by a random draw from a normal distribution with a mean of zero and a standard deviation of .05 at each time point representing an error term in Eq 2. The magnitude of the standard deviations resulted in a ratio out of the total standard deviation observed in Y across the 1000 points in time.

As a demonstration of the proposed integration technique, Fig 2 shows the set points from a single random forest regression model applied to the simulated time series shown in Fig 3. Differences in Y from one data point to the next were treated as the dependent variable and Y, A, and B were treated as the predictors with no specific set of relationships included between Y, A, and B. Visual inspection of Figs 1 and 2 reveals that the model recovers the Cusp Catastrophe surface (comparing Fig 2 to Fig 1) and also properly identifies the locations of both attractive and repulsive set points–differentiated by colors in Fig 2. Interestingly, this application of random forest regression also correctly characterized repulsive set points, which are known to be computationally difficult to detect. Repulsive patterns are characterized by data leaving that topological location, resulting in minimal data over time representing that topological locale. However, the machine was sensitive to the divergence predicting set points and diverging values from those set points.

Table 2 summarizes the simulations. We ran 500 samples in each cell stratified across three time series lengths (250, 500, and 1000) and three different error standard deviation magnitudes (.05, 1, and 1.5). The error magnitudes (prop error in Table 2) corresponded to an average of 13%, 25%, and 34% of variability being due to noise respectively to test recovery under noisy systems akin to how this technique might be applied to biomedical data. For each time series, 18081 points were evaluated as potential set points. The table includes the mean and standard deviation across simulations in the number of set points identified as attractive (# Attractors), repulsive (# Repellers), and transient (# Transient) along with the mean and standard deviation in R square multiple for a linear regression using Y, A, and B as predictors (Linear R^2^) and a Cobb’s pseudo R square specifically designed for detecting the Cusp Catastrophe model (Cusp R^2^). Since the goal of examination of the cusp model is to determine how well the model is able to characterize the complex cusp topology, rather than the specific training and generalizability of the algorithm, we chose to not split the data.

**Table 2:** Summary of simulations.

N	SD Error	Prop Error		# Attractors		# Repellers		# Transients		Linear R^2^		Cusp R^2^	
		Mean	SD	Mean	SD	Mean	SD	Mean	SD	Mean	SD	Mean	SD
250	0.05	0.130	0.009	504.128	100.933	223.020	530.195	1345.132	635.731	0.806	0.056	0.893	0.058
500	0.05	0.131	0.006	533.980	82.331	125.652	327.140	1021.072	427.862	0.817	0.051	0.903	0.059
1000	0.05	0.131	0.004	553.742	54.780	54.538	167.779	749.626	245.422	0.817	0.045	0.913	0.054
250	0.1	0.247	0.020	527.190	93.628	115.836	386.175	1522.014	525.669	0.789	0.061	0.843	0.091
500	0.1	0.246	0.017	563.478	75.272	71.714	242.862	1157.576	356.667	0.799	0.055	0.846	0.083
1000	0.1	0.247	0.008	576.678	55.265	26.118	78.474	877.246	178.464	0.785	0.055	0.830	0.089
250	0.15	0.339	0.030	561.680	92.642	56.426	224.491	1681.170	423.527	0.761	0.073	0.769	0.116
500	0.15	0.340	0.019	592.498	72.755	31.570	97.882	1294.518	257.790	0.753	0.068	0.756	0.113
1000	0.15	0.340	0.020	600.639	54.922	26.701	36.659	1013.108	168.295	0.743	0.056	0.738	0.100

Only a small portion of evaluated points within the random forest models qualified as set points with the vast majority of those qualifying as a set point being categorized as transient followed by attractors, and then repellers. Repulsion should be rare given that few areas and combinations of A and B generate repulsion within the cusp catastrophe. Further, longer time series and those with more error identified fewer transient and repulsive set points though this did not affect attraction in the same manner. The smaller standard deviations as sample size increased was consistent with the longer time series having the opportunity to explore more of the cusp catastrophe, leading to a more consistent number of set points estimated. While the time series showed R-square values at zero, the set points instead followed the cusp model even under high error magnitudes. As a set, this suggests a high degree of recovery for the cusp catastrophe with the many points under which no discernible change was predicted and their categorization as a way to depict the system dynamics.

**Fig 4:**
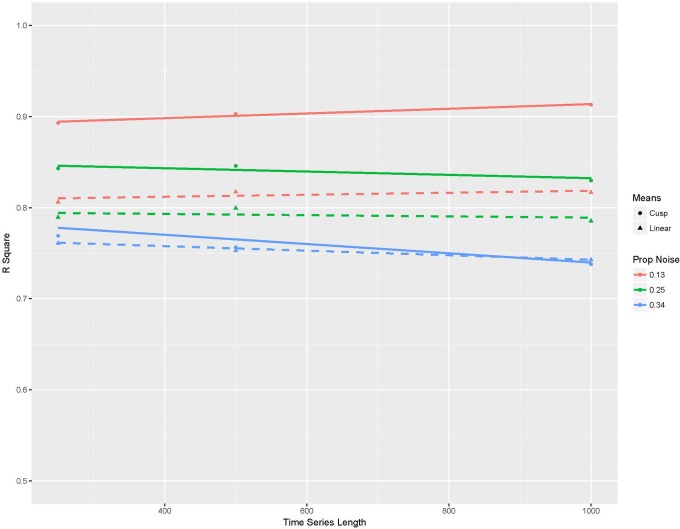
Summary plot of R-square values applied to the set points from random forest models applied to Monte Carlo simulations of the cusp catastrophe. In each case, the set points were extracted by identifying when a predicted change was zero between the 40^th^ and 60^th^ quantiles.

Fig 4 summarizes the average R square values from the Monte Carlo simulations with the R square value on the Y-axis and time series length on the X-axis. Lines are differentiated by the type or R square (least squares and Cobb) and the proportion of noise used to generate the data from Eq 3 (manipulated by the magnitude of the variance in e). As noted, all values are on the extracted set points from the random forest machines only, and when these R square calculations are applied to the raw simulated data, the R square values are zero (for both a least squares linear regression and Cobb’s cusp method). The set points from the random forest models are highly similar to the cusp catastrophe at all time series lengths and levels of perturbation error. In most cases, they are better described as a cusp catastrophe model than as a linear association. However, it is also clear that the combination of the random forest model and focusing on the set points does a better job recovering the cusp catastrophe when perturbation error is small and time series are long in comparison to the other circumstances.

## Exploring topology in a real world example

We now demonstrate the proposed technique using real-world data where the underlying system topology is unknown. The data in our example is taken from a publically available mHealth dataset (https://archive.ics.uci.edu/ml/datasets/MHEALTH+Dataset) that contains passive sensor data collected from participants while they performed several different physical activities (e.g., standing, sitting, walking, climbing stairs, etc.; [50, 51]). Our analyses focus on data collected by two sensors, the Y-axis of an accelerometer worn on the right arm and the Z-axis of an accelerometer worn on the left ankle, for one participant while performing two exercises: walking and climbing stairs. We selected these particular data for an example because they allow for modeling the gait of the individual while performing the exercises. Different gaits represent qualitatively different dynamics [52], and movement serves as a canonical example of an adaptive system, where different contexts (e.g., terrains or exercises) facilitate different gait dynamics [53].

Separate Random Forest models were run for each accelerometer variable (Y-axis on the right arm and Z-axis on the left ankle) where change over time in each accelerometer was predicted by the current value of both accelerometers. The data were collected at 50Hz for a duration of one minute in each activity, totaling 6144 observations. A 50% split was used to generate training and test datasets. The degree to which the Random Forest solution captures the qualitatively different topological features of both exercises is evaluated by a) the R^2^ values of the random forest compared to a pair of linear regressions including both accelerometer values predicting the changes, b) identifying set points and c) seeing the extent to which the identified set points corresponded to different predicted dynamics for the two exercise conditions.

### Results of real-world example

Table 3 provides R^2^ values for the Random Forest model for the training and test sets for the two accelerometers along with R^2^ values for the equivalent linear regressions. In all cases, the Random Forest model was a marked improvement over the linear equivalent. As with the cusp catastrophe model, the set points for the system dynamics were identified using quantiles from the Random Forest model. In this case, twenty-four quantile ranges were examined using the even numbered quantiles (2^nd^ and 98^th^, 4^th^ and 96^th^, etc.). This procedure generated up to twenty-four instances in which a given point can imply no change (i.e., the quantiles include 0) as a function of the Quantiles. Since there were two Random Forest models (one where change in the arm was the outcome and another where change in the ankle was the outcome), set points were defined in both equations simultaneously. To imply a set point requires both equations to contain zero within a given confidence interval for a given arm/ankle coordinate.

**Table 3:** R^2^ values for accelerometer example.

	Training Sample		Validation Sample	
	Least Squares	Random Forest	Least Squares	Random Forest
**Left Ankle**	0.254	0.597	0.216	0.591
**Right Arm**	0.067	0.171	0.069	0.114

**Fig 5:**
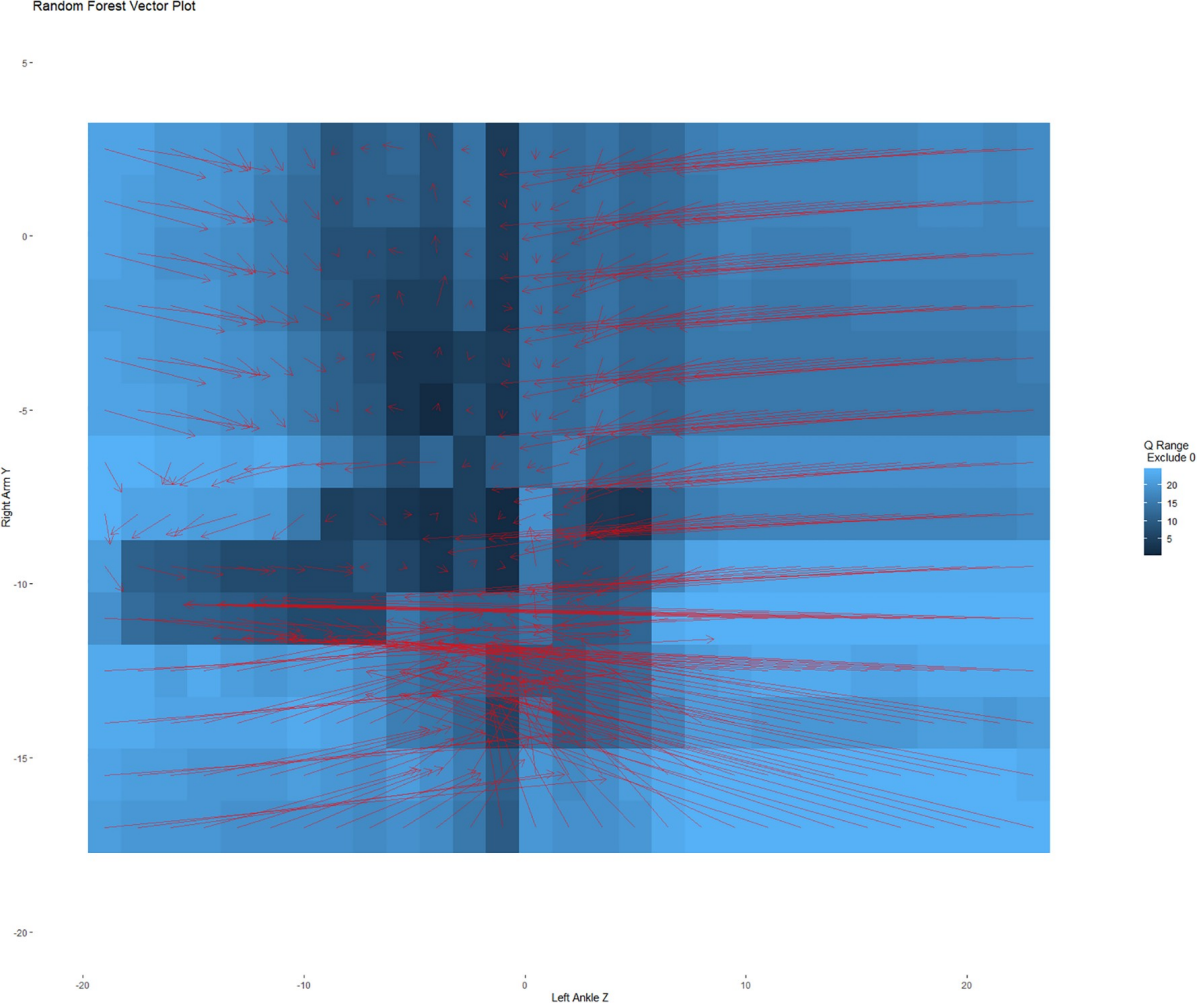
Heat map of possible set points with a predicted vector plot overlay. The heat map shows the number of quantile ranges (using just the even integers) that exclude zero. Darker areas identify coordinates that are likely set points. The vectors are the predicted changes taken from the random forest models.

Fig 5 contains a heat map of the number of quantile ranges that excluded zero change in both equations simultaneously. Darker areas are more likely set points. As one can see from the heat map, a different choice for the quantiles would identify a different number of set points. One point would always be defined as a set point, which is the darkest spot on the heat map (near zero in the Z-axis for the ankle and near -10 for the Y-axis for the arm). A couple of set points are dependent upon the chosen quantiles. For example, the point near -10 in both Y and Z often included zero in both pseudo-confidence intervals, but also had some confidence intervals where at least one of the quantile ranges excluded zero. These two likely set points are highlighted due to their correspondence to the climbing and walking conditions.

Fig 5 also overlays a vector plot. Each arrow represents the predicted change in value from one time point to the next based on the results of the Random Forest models. These flows indicate where data is predicted to move over time, with the set point being the coordinate on which the arrows converge. The technique of identifying the topological behavior of a set point is visually equivalent to examining the predicted arrows closely surrounding a given set point. When the arrows converge towards the point, they indicate attraction. Arrows diverging from a set point indicate repulsion (it is possible to be repulsive and attractive simultaneously).

**Fig 6:**
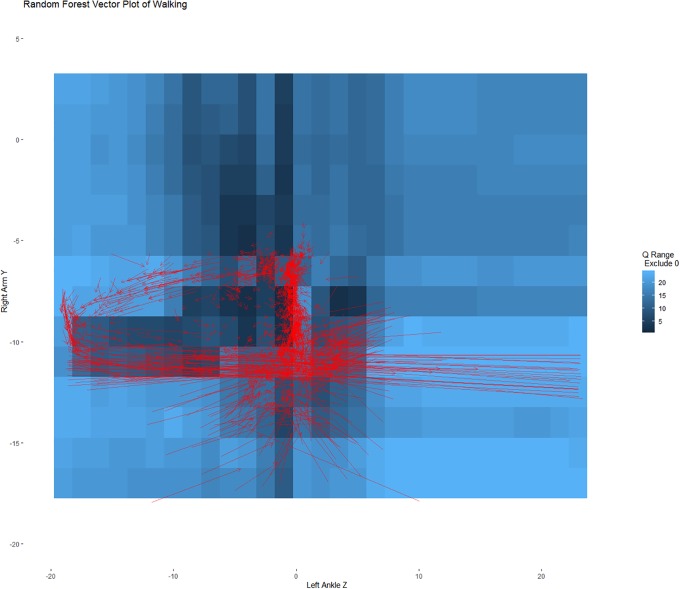
Heat map of possible set points with predicted vector plot only showing values from the validation sample that were in the climbing condition. The predicted vectors in the climbing condition primarily point towards the likely set point approximately at (0,-10) moving towards the point. This is consistent with attraction.

**Fig 7:**
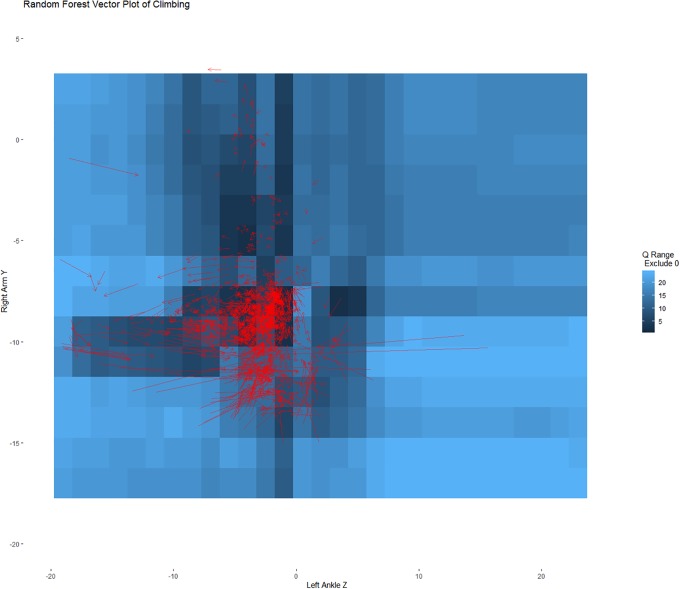
Heat map of possible set points with predicted vector plot only showing values from the validation sample that were in the walking condition. The predicted vectors in the walking condition primarily move around the likely set point approximately at (-10,-10). This is consistent with cycles.

To help evaluate the set points and our ability to recover the dynamics of the system, Figs 6 and 7 show these same predicted changes, separated by when the person was climbing and walking. These figures illustrate the predicted values for the training data rather than showing all possible vector combinations within a range of values illustrated in Fig 5. In Fig 6 (the climbing data), the vast majority of predicted changes are all in relation to the hypothetical set point at (0,-10) showing attraction towards the set point. In Fig 7 (the walking data), the predicted changes all cycle around the set point near (-10,-10). That is, the set points distinguish the different movement dynamics as one would desire, and by examining the set points we can decompose the dynamics.

As a set, these results imply that focusing on local extraction of the set points and stability around those set points is a viable method for describing the system. Combining this approach with machine learning allows for describing the system locally and globally without the reliance on a global set of equations. Given the focus on local interpretation, it is reasonable to expect the results to function in both systems governed by global equations and adaptive systems.

## Discussion

This application of machine learning to dynamical systems proffers two major benefits. For the dynamical systems literature, it provides an exploratory approach to both identify and visualize nonlinear dynamic topologies. This exploration provides information on the complex interactions of variables over time, and through plotting the topology, can serve as an alternative method for identifying the canonical dynamic system, since the canonical systems produce recognizable topological surfaces, with specific features. Further, it allows for quantification and investigation of set points and local behaviors of data around those set points from the predicted random forest regression models.

As a tool, extracting set points, and the local behaviors around those set points greatly reduces what is needed to understand the predictions by a machine because this approach divides the prediction space into regions of behavior. Basins of attraction, repulsion, and other system patterns can be identified and combined to demonstrate where the machine would predict data to go over time. Such time evolution depictions generate a map as to where a machine would predict the system to go over time and where the machine would predict unexpected behavior based on a global equation characterizing the system. Thus, it can be diagnostic in the degree to which the observed system conforms to one described by a global equation.

As noted earlier, not all systems are expected to conform to global equations and may only instead display localized dynamics. Under such systems (e.g., complex adaptive systems) this technique provides a novel way to understand their behavior without requiring a global equation form to express it. When used in this way, a visualization of the topological map of the system as it adapts over time, or under certain different control parameters can be generated. This approach can map the canonical or adaptive system form by seeking localized descriptions of the topology, which may diverge from a canonical solution entirely.

Further, the cusp catastrophe serves as an ideal dynamic system for testing the functionality of the proposed method due to its complex topological feature set. The system demonstrates attractors and repellers and multi-stability. The machine was able to accurately recapture all these components of this system. Repellers are of particular interest since they are sparse on data and difficult to identify. Machines excel at dealing with sparse data, and this technique demonstrates the technique’s robustness in recapturing the topology of the system in spite of the minimal data representing particular topological features.

The accelerometer example strengthens the argument as to the utility of the technique. The example illustrates how, through two simultaneous equations, the designated set points corresponded to behavioral differences in walking and climbing stairs. Notably, no information as to which physical activity was included in the models. And yet, set point differences corresponded to the activities with very different dynamics predicted within those basins of activity. This is consistent with our argument that this approach can be used to differentiate the dynamics that underlie a system and provide a means to visualize and explore the resultant model.

### Limitations

Though we argue that the approach applies to adaptive systems, we primarily validated the approach with a canonical global equation. Our choice of using the cusp catastrophe was driven by the equation’s known flexibility akin to what is commonly believed to be part of adaptive systems. The cusp catastrophe is known to exhibit all five properties associated with catastrophic change where only some basins are stable and only some of the time [44, 54]. Thus, it creates a hard test for extracting dynamical systems properties. The proposed procedure’s ability to extract such a system dynamic is a testament to its robustness. Simultaneously the cusp catastrophe model is, like many canonical dynamic systems, represented by a simple equation form that has been commonly applied to many areas across science. Thus, it helps support our claim that the approach should be useful to both physical and adaptive systems. However, this statement is based on assumptions about the functionality of adaptive systems themselves.

Further, in our exploration of the cusp catastrophe model, the technique demonstrated the ability to recover the system’s topology with long and short time series. It is not yet known at what point a time series will be too short to properly recover the system’s dynamics and this requirement should be a function of the system complexity (i.e., more complex systems should require longer time series). Cases where the R-square values from the linear and cusp based calculations were indistinguishable are circumstances where the proposed approach lost sensitivity of the cusp itself. This result suggested that there are likely to be circumstances where the necessary nuance for a complex system may not be fully captured.

A similar limitation is related to the role of data sparsity. Since the approach capitalizes on localized estimation, it is possible for a time series to lack data that represents a basin or local set of topological features. The ability of the proposed approach to recover repellers suggests that the model is robust to this limitation under some circumstances.

Another limitation of the proposed approach involves the computational time required. Identification of sets points dramatically increases computational demands in large datasets. In our simulation, we built a grid of points and explored each as set points. Such an approach is not feasible for large data sets with many variables. However, it is possible to decrease computational demands by capitalizing on the same systems properties as are being extrapolated. For example, attraction is defined by data approaching the set point over time. Thus, one could start at a random point and iterate forward until minimal change occurs similar to Maximum Likelihood estimation. Exploration of many starting values would allow for reducing the possible set points, and each point of minimal change could then be explored for its stability making for a more efficient procedure.

Lastly, we chose to rely on a difference equations approach rather than differential equations. Translating the approach illustrated above to differential equations is possible and would require integrating machine learning with a derivative estimation technique. Doing so would allow for capitalizing on approaches based on stochastic differential equations [55], where one set of equations is specified for the dynamics, and a different series of equations is specified for the stochastic prediction of values. Under such a specification, the distribution of the stochastic elements of observed values could be sampled from as a way to capture variability for set points, rather than quantile regression. This extension is only one example, and there are many possible ways to integrate machine learning techniques and differential equations that deserve further exploration.

### Future directions

Not only can this technique easily integrate with differential equations in the future, but it could also be extended to examine multivariate systems with the accelerometer example being a simple two-dimensional version. As described above, this extension would require vectorizing the outcomes and predictors. Set points in these models would exist in as many dimensions as there are equations rather than one. This approach would yield the multivariately stable locations in the topological map, and any number of simultaneous outcomes could be examined in this fashion applying the same logic.

Cycles and chaotic attractors have at least one dimension in which the system neither approaches nor diverges from the related set point, but where change occurs angularly relative to the set point instead. For example, a cycle was observed with one of the set points in the walking data. This circumstance creates a more complicated scenario for identifying the behavior around the set point as points would predict motion angular to the set point rather than towards or away. However, just as a single equation can be generalized to identify set points as locations where all the predicted changes are zero, so too can the identification of the type of topological feature. In this case it is possible to estimate a Jacobian matrix (matrix of partial derivatives) around each set point by extracting the predicted changes slightly above and below each set point circling the set point and calculating least squares slopes as a function of the values used to go slightly above and below the set point predicting these changes. The slopes constitute the Jacobian matrix, and the characteristic roots/eigenvalues of this matrix of slopes identify the dynamic nature of the topological features [56].

By expanding machine learning to identify the set points and information on the topological features that they represent, a map can be generated of where time series is predicted to go next given where it currently is. This integration provides a qualitative description of the model results as well as details in regard to the complexity of the model. Complexity of the model is represented through identification of accessibility and multimodality as a function of which variables differentiate or fail to differentiate the topological features. Further, the importance of a variable can be redefined in terms of accessibility of the various dynamic features. For example, in the cusp catastrophe, the importance of A and B are stipulated by their ability to stabilize and change the set points. As shown in Fig 2, by plotting the topological predictions from the machine, the accessible and inaccessible locations of the system can be examined. Visual inspection reveals when one or multiple stable states are available, and how stable those states are, as a function of the control parameters, A and B. Local definitions of what patterns are desirable can be identified and then the importance of a specific variable can be assessed in terms of its relation to the feature’s accessibility.

In addition, it is possible that many deep learning models are already generating or capitalizing on systems solutions. The change as outcome approach illustrated in Eq 2 is a simple transformation of one where the future value of an outcome is predicted as a function of current value or past value and other predictors (an autoregressive relationship; [57]). In these cases, the machine learning solutions are already generating models consistent with a dynamical systems equation, but not providing specific information on the topological features. By applying methods to assess local dynamics–identifying the set points and the behavior local to the set point–one is able to peer into very complicated machine learning solutions, drawing out fundamental descriptions of the topology as well as where the system is predicted to go next.

### Summary

Both machine learning and dynamical systems already have bright futures for the expansion of science by providing road signs for the meaning and prediction of complex outcomes. Through this general integration, both are fundamentally improved upon. The machine learning tools provide an approachable methodology for exploring data consistent with nonlinear dynamic models but without the necessity of stipulating a specific nonlinear model. Thus, it becomes a general exploratory approach for the systems scientist toolbox. For the machine learner, the key principles from systems science provide a way of understanding the principles of what a given machine learning model is generating.

Since the integration is a general approach rather than specific to a type of machine learning tool or systems model, it allows for a general integration of ideas. Systems science explicitly focuses on data over time as its main topic of interest. This type of data is sometimes incorporated into machine learning contexts, though machine learning has generally been less interested in identification of the underlying dynamic rather than correcting for nonstationary or identification of a canonical system. This technique provides a new systems tool, specifically focused on the identification of topological features in data over time.

## Supporting information

S1 File Catastrophe simulator. R code and explanation for simulating time series from a discrete cusp model.
(DOCX)

S2 File Extracting set points and stability. R code and explanation for extracting set points from the discrete cusp model.
(DOCX)
