# Supplementary material for: Ghost hunting in the nonlinear dynamic machine
Source: PLoS One. 2019 Dec 19;14(12):e0226572. doi: 10.1371/journal.pone.0226572 (PMC6922341; doi:10.1371/journal.pone.0226572)
Supplement: S1 File — R code and explanation for simulating time series from a discrete cusp model. (DOCX) [file pone.0226572.s001.docx]

## **Catastrophe simulator**

The following code in R generates a time series of a specified length (length) with an initial value of the time series (initial). The cusp catastrophe has two different control parameters A & B. The asymmetry parameter (A) specifies differential stability to higher or lower values of Y. The bifurcation parameter (B) specifies for the possibility of one or three separate set points. Multistability within the cusp occurs when B is positive and A is close to zero.

The formulation used in the simulator is a discrete form of the cusp catastrophe model that ignores some constants used in translating the cusp formula. These limit the range in which the formulation will function with some possible values being able to generate impossible time series that go to infinity. If this occurs, the simulator will provide a warning that the values of Y went out of bounds.

The simulator defaults to sampling A and B from a uniform distribution between -.5 and .5. It is also possible to instead fix A and B to a constant (through changing the fix variables to true and providing values for A and B). This allows for exploration of a specific space in the surface rather than the entire range of the cusp model.

Finally, perturbation magnitude is the standard deviation of the perturbations or error term in predicting change for each point in time. A value is drawn from a normal distribution conforming to this standard deviation and added at each point in time prior to calculating the next estimate (carrying forward the error term and thus consistent with the notion of perturbations or dynamic error). Also a seed value can be specified to reproduce an identical time series. It is possible that certain combinations can build a time series that goes out of bounds. If this happens, it rebuilds the time series with a different seed value. This will generate code warnings.

cusp_gen<-function(Fix_A=FALSE,Fix_B=FALSE,A=0,B=0, length=1000,initial=rnorm(1,0,1),perturbation_magnitude=.1, seed=NULL){

count<-1

repeat{

if(is.null(seed)==TRUE){

seed_t=round(runif(1,0,1000000000))

}else {seed_t=seed}

set.seed(seed_t)

data_perturb<-matrix(,nrow=length+1,ncol=5)

data_perturb[,5]<-rep(seed_t,times=nrow(data_perturb))

data_perturb[,1]<-seq(from=1, to=length+1)

#A and B are are normally distribution with means of .1 and sd of .25

if(Fix_A==FALSE){

data_perturb[,3]<-runif(nrow(data_perturb),-.5,.5)

#data_perturb[,3]<-rnorm(nrow(data_perturb),0,.25)

}else{

data_perturb[,3]<-rep(A,times=nrow(data_perturb))

}

if(Fix_B==FALSE){

data_perturb[,4]<-runif(nrow(data_perturb),-.5,.5)

# data_perturb[,4]<-rnorm(nrow(data_perturb),0,.25)

}else{

data_perturb[,4]<-rep(B,times=nrow(data_perturb))

}

data_perturb[1,2]<-initial

for(i in 1:length){

current<-i+1

old_y<-data_perturb[i,2]

data_perturb[current,2]<-old_y+rnorm(1,mean=-old_y^3+data_perturb[i,4]*old_y+data_perturb[i,3],sd=perturbation_magnitude)

}

if(is.nan(data_perturb[length+1,2])==T){

if(count==50){print("Time Series Went Out of Bounds");

break

}

count<-count+1

print("Time Series went out of bounds, trying different seed")

}else{

break

}

}

data_perturb<-as.data.frame(data_perturb)

names(data_perturb)<-c("time","y","A","B","seed")

return(data_perturb)

}
