# Supplementary material for: Ghost hunting in the nonlinear dynamic machine
Source: PLoS One. 2019 Dec 19;14(12):e0226572. doi: 10.1371/journal.pone.0226572 (PMC6922341; doi:10.1371/journal.pone.0226572)
Supplement: S2 File — R code and explanation for extracting set points from the discrete cusp model. (DOCX) [file pone.0226572.s002.docx]

## **Extracting set points and stability**

The following code in R uses randomForestSRC to generate a random forest machine based on the discrete difference as the outcome and, Y, A, and B as the predictors. To do so, the data from the simulator is modified to include a discrete difference (lead minus current) in Y and this difference is the outcome. Once the machine was generated we then build a grid of values in Y, B, and A. The machine is then used to generate predicted values at the 40^th^ and 60^th^ quantiles based on the random forest/quantile regression integration. If zero is between the quantiles, the combined YBA vector is retained as a set point. Set points are then evaluated for their stability by generating predicted values at the same B and A, but where Y was .05 above and below the set point value. At the end of the code we draw the results.

#Generate the cusp data

data<-cusp_gen()

#Calculate leads and differences

require(Hmisc)

data$y_1<-Lag(data$y, -1)

data$y_diff<-data$y_1 - data$y

#Run the Random Forest Model

require(randomForestSRC)

#The call for a standard random forest model

p1.rf<-rfsrc(y_diff ~ y + A + B,data=data)

#And the Quantile model, do not need both if retain .5 quantile too

p1.qrf<-quantreg(y_diff ~ y + A + B,data=data,prob=c(.4,.6))

#Build a grid of predicted changes

temp<-data.frame(expand.grid(y=seq(-1,1,.05),A=seq(-.5,.5,.05),B=seq(-.5,.5,.05)))

#Prediction space of grid, 18081 points in total

pred1<-predict(p1.rf,newdata=temp,se=FALSE)

temp$pred1<-pred1$predicted

#Due to a change in randomforestSRC, cannot run all 18081 points at once in quantile reg

temp1<-temp[1:5000,]

temp2<-temp[5001:10000,]

temp3<-temp[10001:15000,]

temp4<-temp[15001:18081,]

#Build predicted quantiles

p1.rf.q1<-quantreg(object=p1.qrf,newdata=temp1,prob=c(.4,.6))

p1.rf.q2<-quantreg(object=p1.qrf,newdata=temp2,prob=c(.4,.6))

p1.rf.q3<-quantreg(object=p1.qrf,newdata=temp3,prob=c(.4,.6))

p1.rf.q4<-quantreg(object=p1.qrf,newdata=temp4,prob=c(.4,.6))

#Define setpoint as 0 is between predicted quantiles

temp1$q_sig[0>=p1.rf.q1$quantreg$quantiles[,1] & 0<=p1.rf.q1$quantreg$quantiles[,2]]<-0

temp1$q_sig[is.na(temp1$q_sig)==TRUE]<-1

temp2$q_sig[0>=p1.rf.q2$quantreg$quantiles[,1] & 0<=p1.rf.q2$quantreg$quantiles[,2]]<-0

temp2$q_sig[is.na(temp2$q_sig)==TRUE]<-1

temp3$q_sig[0>=p1.rf.q3$quantreg$quantiles[,1] & 0<=p1.rf.q3$quantreg$quantiles[,2]]<-0

temp3$q_sig[is.na(temp3$q_sig)==TRUE]<-1

temp4$q_sig[0>=p1.rf.q4$quantreg$quantiles[,1] & 0<=p1.rf.q4$quantreg$quantiles[,2]]<-0

temp4$q_sig[is.na(temp4$q_sig)==TRUE]<-1

temp$q_sig<-c(temp1$q_sig,temp2$q_sig,temp3$q_sig,temp4$q_sig)

#Shift the grid up and down by .05 value of

#.05 is arbitrary to move around the set point

temp_a<-data.frame(expand.grid(y=seq(-.95,1.05,.05),A=seq(-.5,.5,.05),B=seq(-.5,.5,.05)))

temp$above<-predict(p1.rf,newdata=temp_a,se=FALSE)$predicted

temp_b<-data.frame(expand.grid(y=seq(-1.05,.95,.05),A=seq(-.5,.5,.05),B=seq(-.5,.5,.05)))

temp$below<-predict(p1.rf,newdata=temp_b,se=FALSE)$predicted

#Only retain the set points

temp1<-subset(temp,q_sig==0)

#Define type of topology using what happens .05 above and below set point in Y

temp1$type[temp1$above<0 & temp1$below>0]<-"Attractor"

temp1$type[temp1$above>0 & temp1$below<0]<-"Repeller"

temp1$type[is.na(temp1$type)==TRUE]<-"Transient"

#Draw the set points color coded by type

require(car)

require(rgl)

scatter3d(data=temp1,y~A+B|as.factor(type),surface=FALSE,fit="smooth", point.col=c(1,2,3),sphere.size=2)
